# Supplementary material for: Cell-specific and shared regulatory elements control a multigene locus active in mammary and salivary glands
Source: Nat Commun. 2023 Aug 17;14:4992. doi: 10.1038/s41467-023-40712-0 (PMC10435465; doi:10.1038/s41467-023-40712-0)
Supplement: Supplementary file 1 — Supplementary Information [file 41467_2023_40712_MOESM1_ESM.pdf]

Supplementary information

Cell-specific and shared regulatory elements control a multi-gene locus active in mammary and salivary glands

Hye Kyung Lee[1,*], Michaela Willi[1], Chengyu Liu[2], Lothar Hennighausen[1,*]

[1]Laboratory of Genetics and Physiology, National Institute of Diabetes and Digestive and Kidney Diseases, US National Institutes of Health, Bethesda, Maryland 20892, USA.
[2]Transgenic Core, National Heart, Lung, and Blood Institute, US National Institutes of Health, Bethesda, Maryland 20892, USA.
* Correspondence to: H.K.L (hyekyung.lee@nih.gov) and L.H (lotharh@niddk.nih.gov)

Supplementary Figures

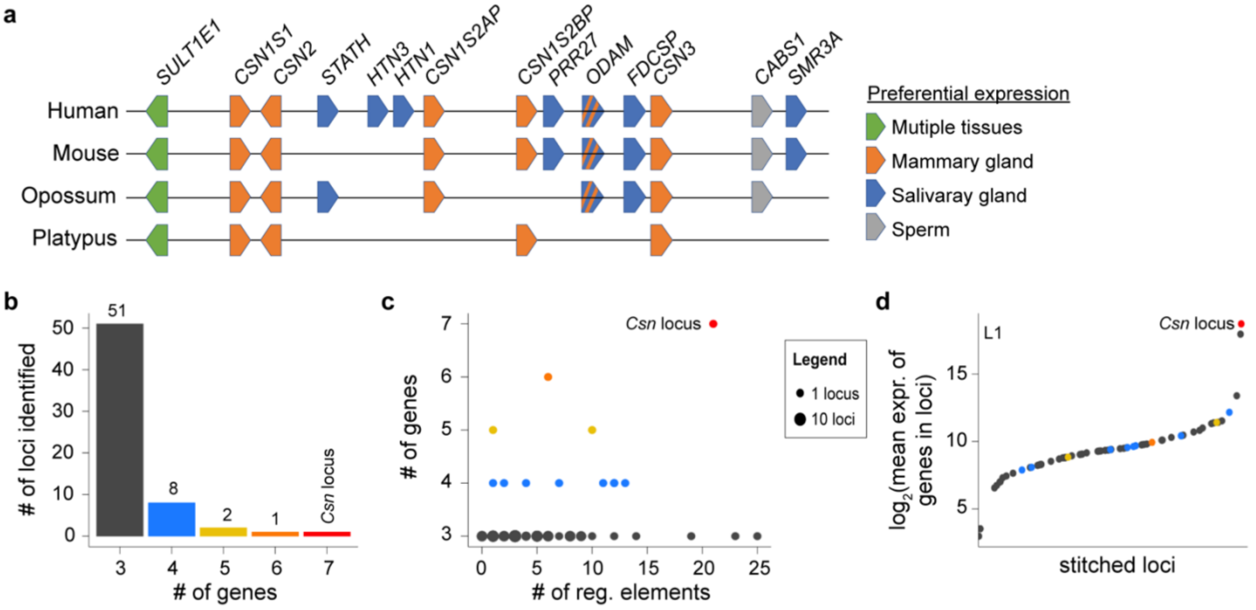

Supplementary Fig. 1. Identification of loci with complex enhancers and stitched genes. a, Diagram presents gene structure within the *Casein* locus in several extant mammalian species. **b,** The bar plot shows the number of genes within multi-gene loci identified by stitching genes induced more than 2-fold between day six of pregnancy (p6) and days one (L1) and 10 (L10) of lactation. **c,** The plot compares the number of regulatory elements to the number of stitched genes in each multi-gene locus. **d,** The casein locus contains the highest expressed genes during pregnancy.

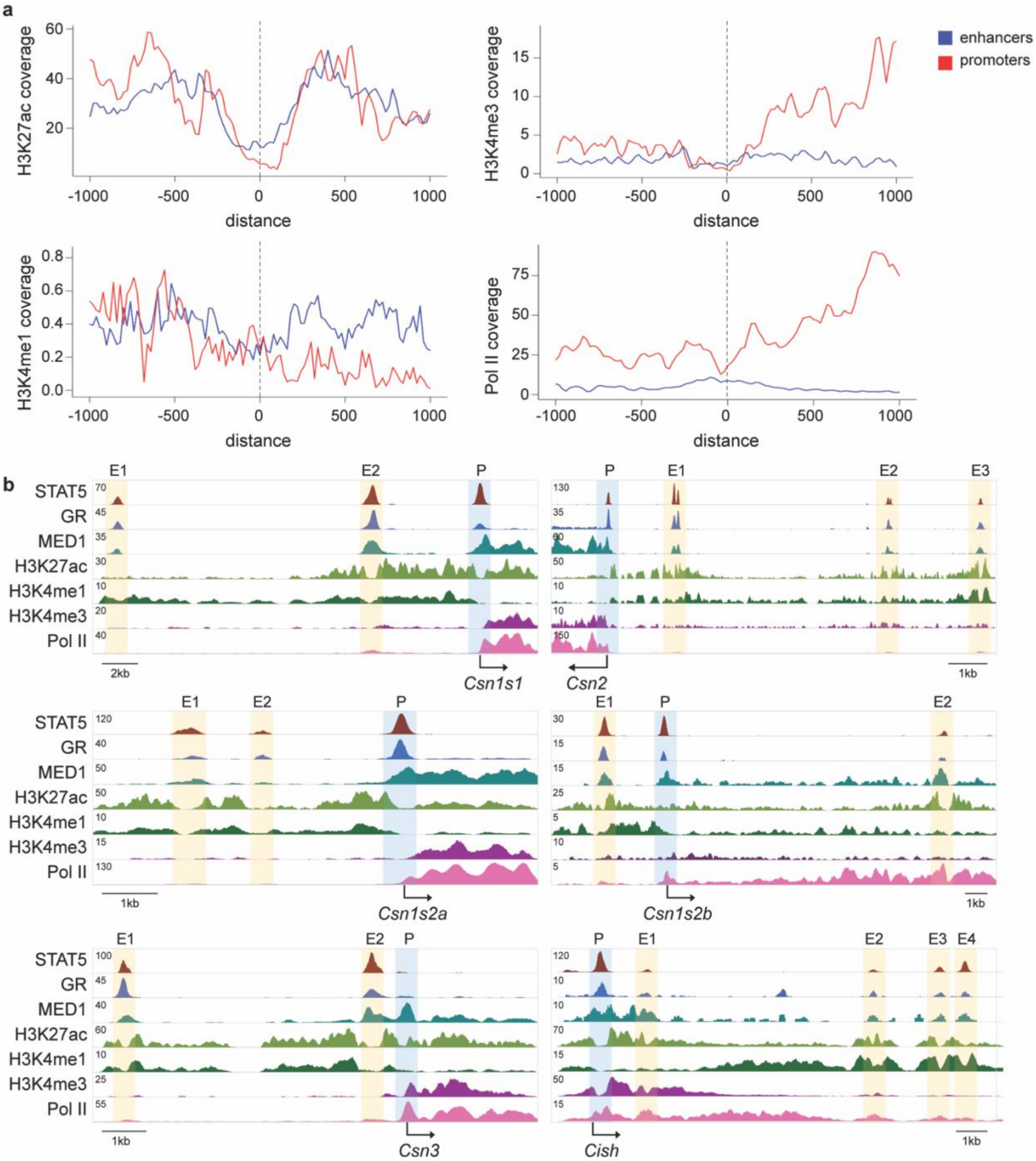

Supplementary Fig. 2 Chromatin features of candidate enhancers and promoters in the *Casein* locus. a, The coverage plots show the patterns of enhancer marks, H3K27ac and H3K4me1, and promoter marks, H3K4me3 and Pol II, within the *Casein* locus (blue, enhancers; red, promoters). Dot lines indicate the center of the peaks. **b,** STAT5, GR, H3K27ac, H3K4me1, H3K4me3 and Pol II landscape displays the regulatory

elements on individual gene loci in the *Casein* locus at day one of lactation (L1). STAT5 peaks on Csn1s1-P, Csn2-P, E1 and E2, Csn1s2a-P, E1 and E2, Csn1s2b-P, E1 and E2, Csn-SE-E3 and Csn-E2 coincide with GAS motifs. The highlighted yellow and blue shades indicate the enhancers and promoters, respectively. The *Cish* locus served as control.

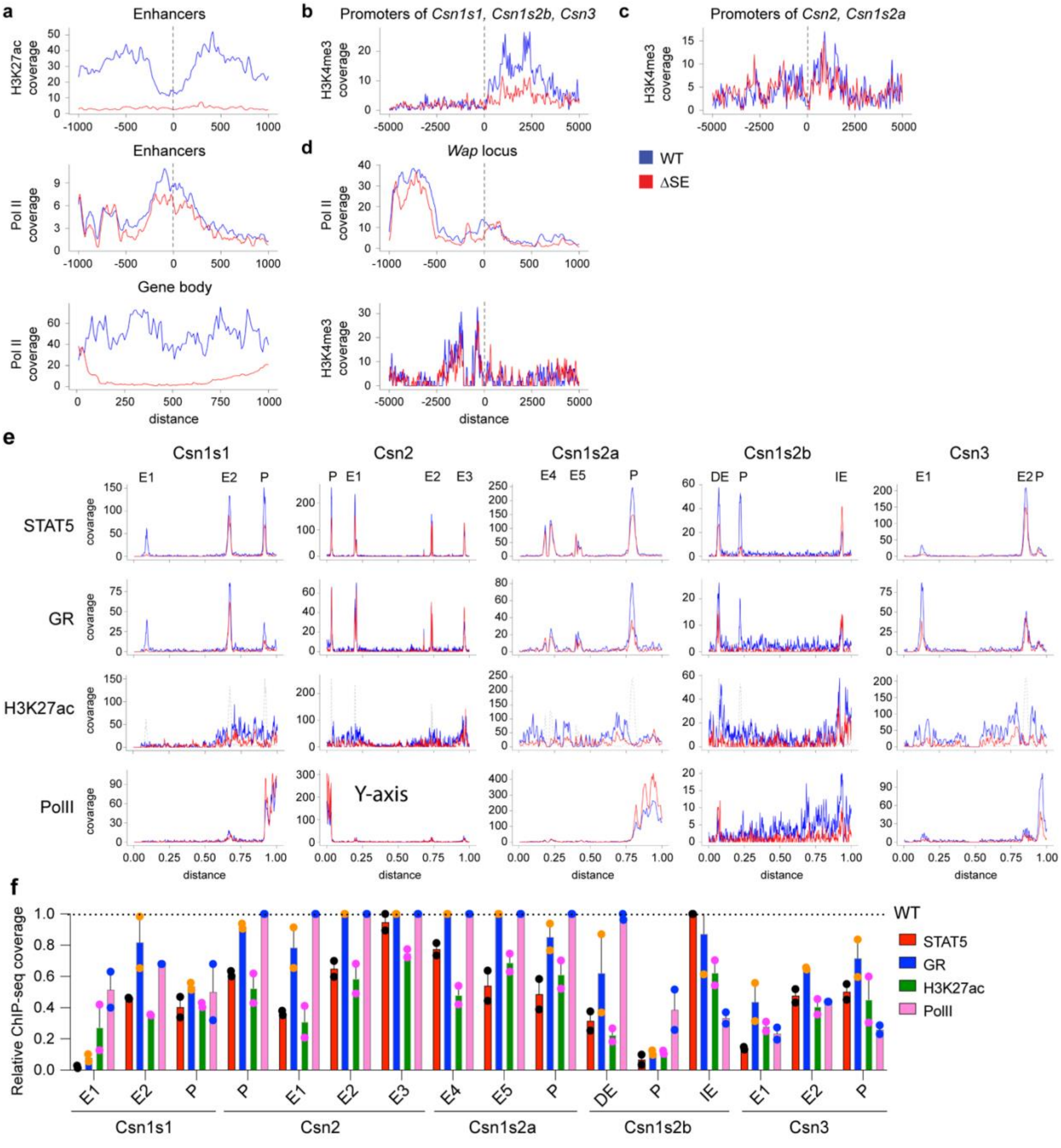

Supplementary Fig. 3 Genomic features of the *Casein* locus upon deletion of the super-enhancer. a, The coverage plots show that H3K27ac and Pol II were strongly reduced at the enhancer elements and gene body in the *casein* locus in lactating mammary tissue (collected within 12 hours post-partum (pp <12h)) (blue, WT; red, mutant). **b,** Coverage plot showing H3K4me3 at the transcription start sites of the *Csn1s1*, *Csn1s2b* and *Csn3* genes was reduced in accordance with the reduction in gene

expression in mutant mice. **c,** Coverage plot showing H3K4me3 at the Csn2 and Csn1s2a genes was equal in WT and mutants. **d,** Coverage plot showing Pol II and H3K4me3 at another milk protein gene, *Wap* gene, was equal in WT and mutants. **e,** Enhancer-promoter coverage of each casein gene locus showing STAT5, GR, H3K27ac and Pol II in WT (blue) and ΔSE mice (red). Gray dot peak in H3K27ac plot presents STAT5 binding regions. **f,** STAT5, GR, H3K27ac and Pol II coverage was calculated after variation between data set was normalized with *Cish* enhancer and promoter coverage, respectively. The coverage was normalized again to WT levels. Results are shown as the means ± SEM of independent biological replicates (n = 2). Source data are provided as a Source Data file.

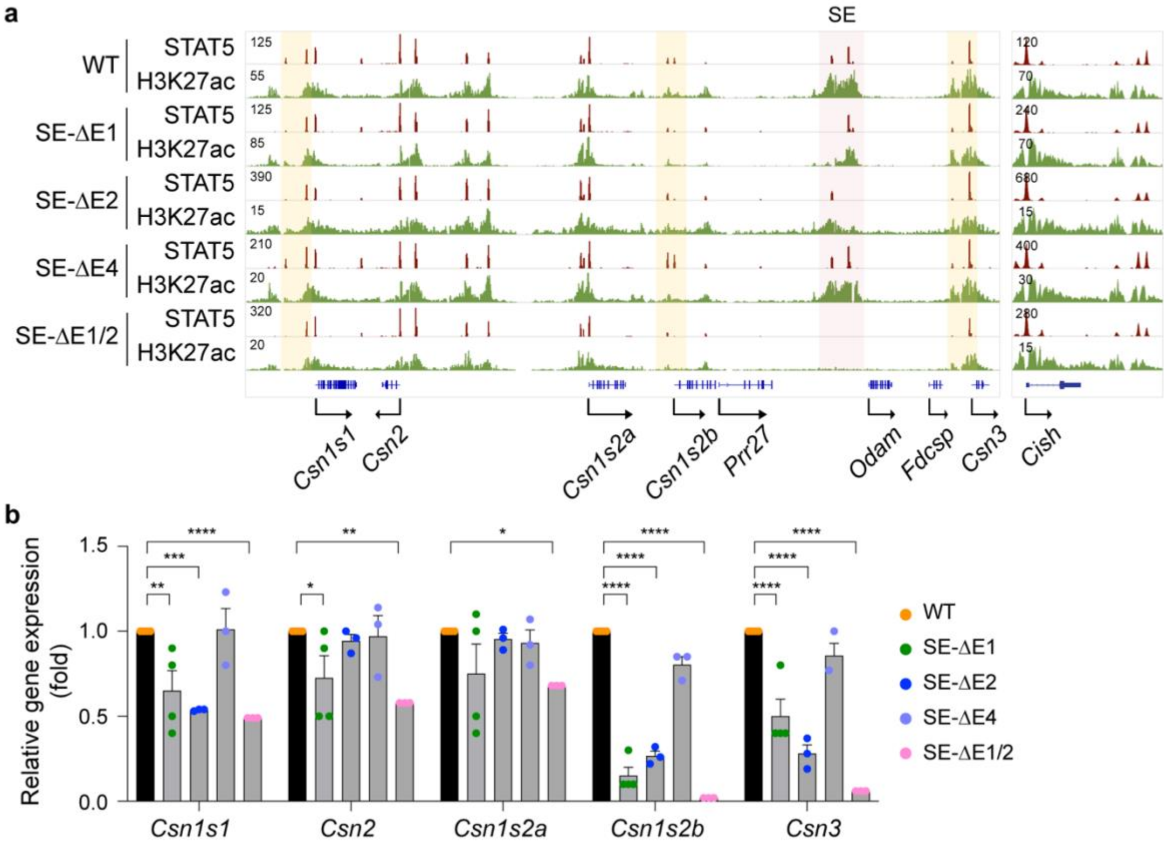

Supplementary Fig. 4 Biological significance of constituent enhancer modules within the super-enhancer. a, ChIP-seq assays confirmed loss of enhancers in mammary tissue from ΔE1, ΔE2, ΔE4 and ΔE1/2 mice at day one of lactation (L1) or within 12 hours post-partum (pp <12h). In ΔE2, E3 and E4 failed to be established. The red and yellow shades indicate the super-enhancer and enhancers, respectively. The *Cish* locus served as control. **b,** Expression of *Csn* genes was measured in mammary tissue of mice carrying individual enhancer deletions by qRT-PCR at day one of lactation (L1) and normalized to *Gapdh* levels (WT, $n = 6$; ΔE1, ΔE2, ΔE4 and ΔE1/2, $n = 4$). Results are shown as the means ± SEM of independent biological replicates. 2-way ANOVA with Dunnett's multiple comparisons was used to evaluate the statistical significance of differences between WT and mutants. $p^{*} < 0.05$, $p^{**} < 0.001$, $p^{***} < 0.0001$, $p^{****} < 0.00001$. Source data are provided as a Source Data file.

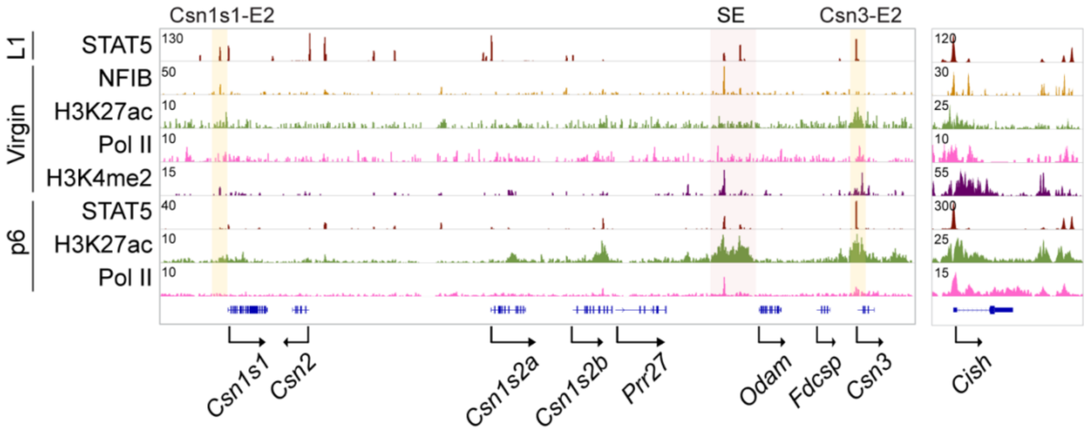

Supplementary Fig. 5 Super-enhancer structures in mammary tissue of virgin and early pregnant mice. Chromatin characteristics of the *Casein* locus were determined by ChIP-seq for STAT5, GR and H3K27ac in WT and ΔSE tissues from virgin and day six pregnant (p6) mice. The red and yellow shades indicate the super-enhancer and enhancers.

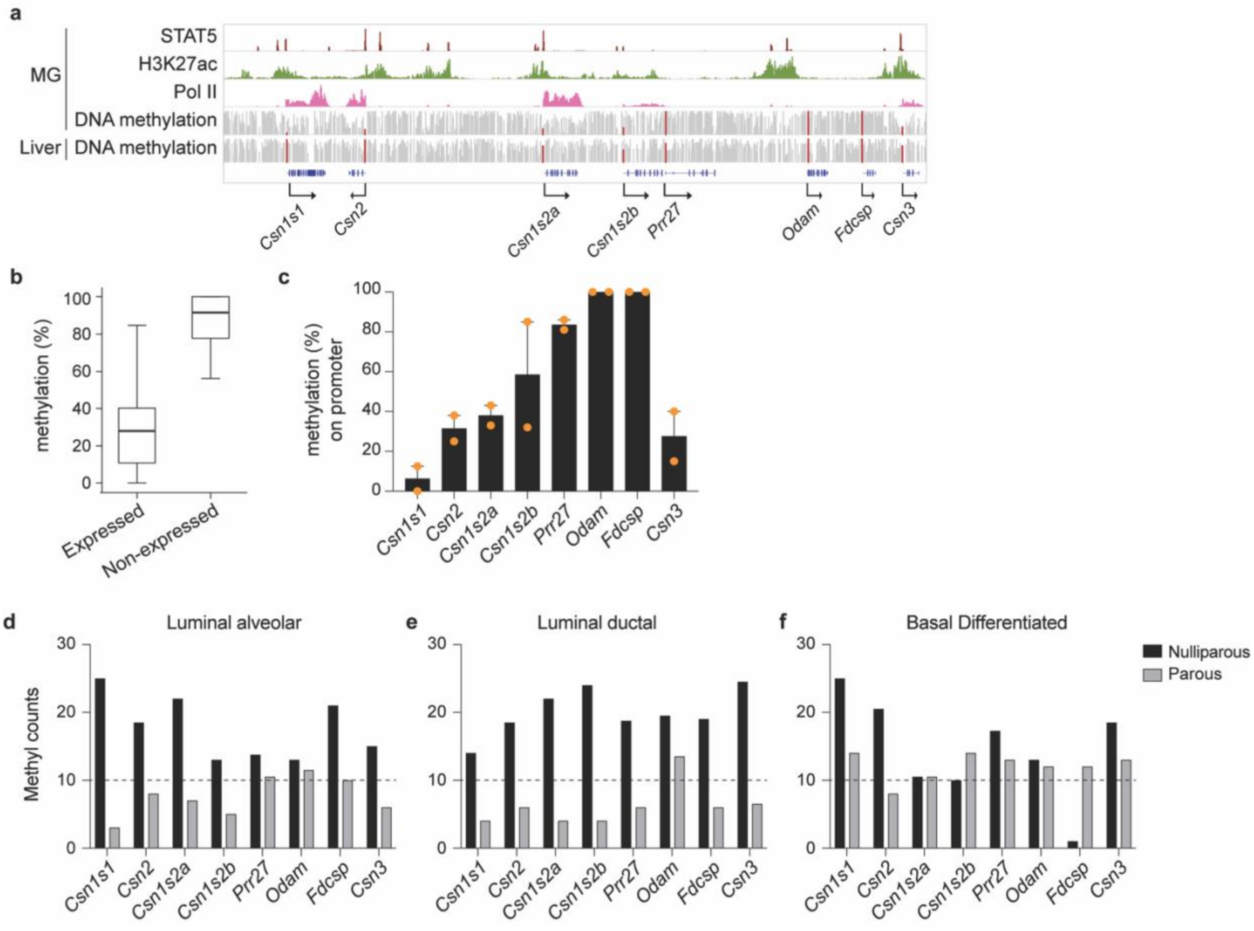

Supplementary Fig. 6 DNA methylation of the *Casein* locus in mammary glands, liver and sorted mammary cells. a, Peaks of DNA methylation reveal inactive and active gene promoters at day one of lactation (L1). **b,** The box-and-whisker plot shows methylation levels of non-expressed and expressed genes in the *Casein* locus in mammary tissue (n = 2). Median, middle bar inside the box; IQR, 50% of the data; whiskers, 1.5 times the IQR. **c,** The bar plot shows methylation level of all genes in the Csn locus in mammary tissues. Results are shown as the means ± SEM of independent biological replicates (n = 2). **d-f,** DNA methylation patterns of casein locus genes in sorted cells from Nulliparous (virgin) and parous mice[21]. Source data are provided as a Source Data file.

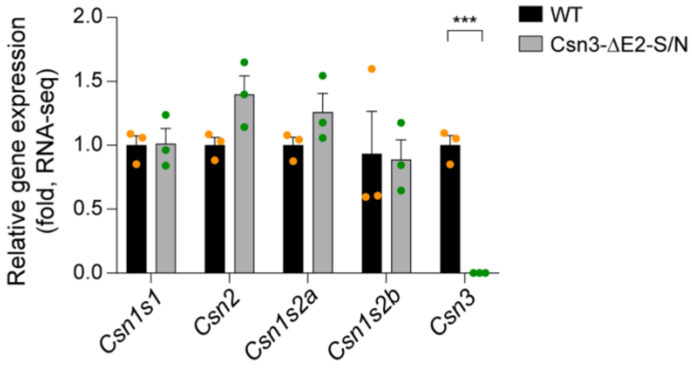

Supplementary Fig. 7 Limited transcriptional activity of the *Csn3* local enhancer. mRNA levels of *Casein* genes were measured by RNA-seq in lactating mammary tissue (day 18 of pregnancy, p18) from WT and mice lacking the *Csn3* proximal enhancer (ΔE2-S/N) ($n = 3$). Results are shown as the means ± SEM of independent biological replicates. 2-way ANOVA with Sidak's multiple comparisons was used to evaluate the statistical significance of differences in WT and mutant. p*** < 0.0001. Source data are provided as a Source Data file.

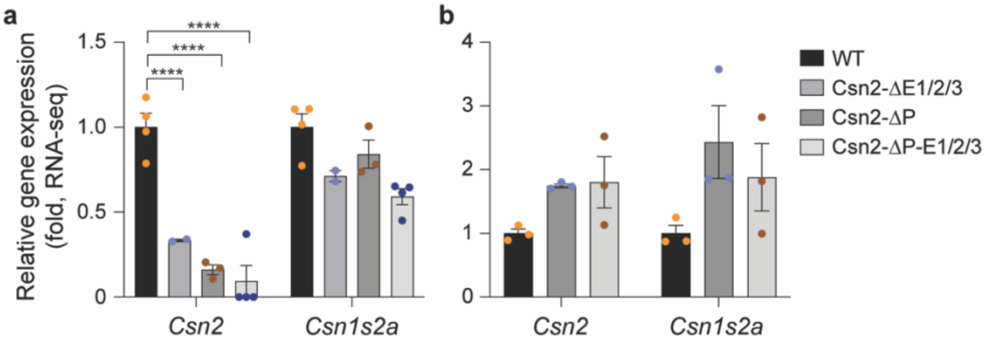

Supplementary Figure 8. Activities of the *Csn2* promoter and enhancers in virgin mice and day 10 of lactation. The *Csn2* promoter-based STAT5 binding sites and the three enhancers are required for gene expression during lactation but not in non-parous (virgin) mice. Expression of *Csn*2 gene was measured in mammary tissue at day 10 of lactation (L10) (a) and in virgin (b) wild type and mutant mice (n = 3) by RNA-seq. Results are shown as the means ± SEM of independent biological replicates. One-way ANOVA with Dunnett's multiple comparisons was used to evaluate the statistical significance of differences between WT and mutants. p**** < 0.00001. Source data are provided as a Source Data file.

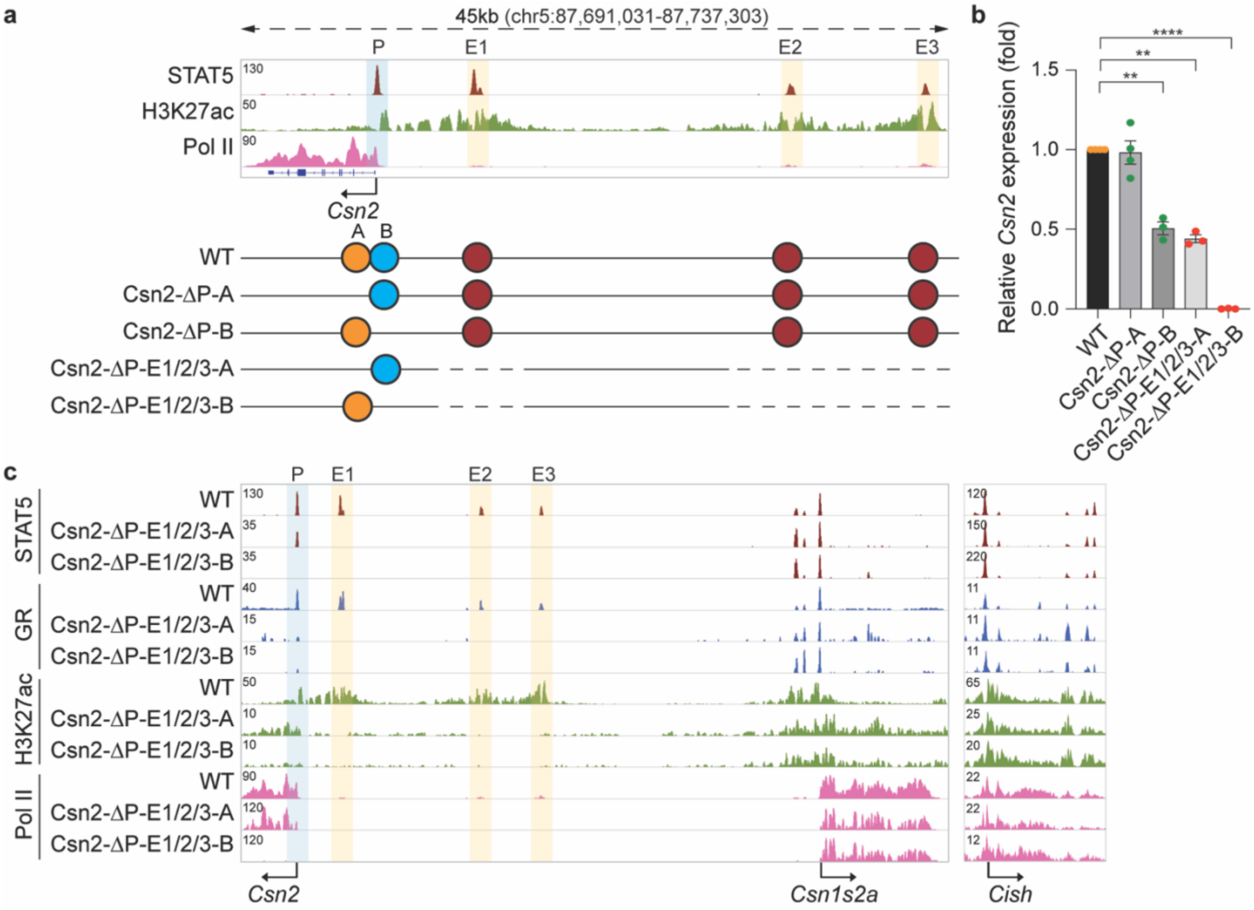

Supplementary Figure 9. The non-canonical, but not the canonical, STAT5 GAS motif is required for *Csn2* promoter activity. a, Diagram of the *Csn2* promoter mutations introduced into the genome of WT and ΔE1/2/3 mice using deaminase base editing. The canonical GAS motif A is shown as orange circles, and the non-canonical GAS motif B is shown in blue. The highlighted yellow and blue shades indicate the enhancers and promoter, respectively. **b,** *Csn2* mRNA levels in lactating mammary tissues from WT and mutant mice at day one of lactation (L1) were measured by qRT–PCR and normalized to *Gapdh* levels. Results are shown as the means ± SEM of independent biological replicates (WT, ΔP-A, $n = 4$; ΔP-B, ΔP-E1/2/3-A, ΔP-E1/2/3-B, $n = 3$). Two-tailed t-test with Welch's correction was used to evaluate the statistical significance of differences between WT and each mutant mouse line. $p^{**} < 0.001$, $p^{****} < 0.00001$. **c,** The *Csn2* locus including *Csn2* promoter was profiled using ChIP-seq in WT and mutant tissue at day one of lactation (L1). The *Cish* locus served as control. Source data are provided as a Source Data file.

Supplementary Tables

Supplementary Table 1. List of multi-gene loci in lactating mammary tissue.

chr	start	stop	number of genes	genes
chr1	13564693	14001780	3	Lactb2, Tram1, Xkr9
chr1	16641725	16715933	3	Ly96, Tceb1, Tmem70
chr1	37430172	37719811	3	2010300C02Rik, Mgat4a, Unc50
chr1	91179822	91321080	3	Ramp1, Scly, Ube2f
chr1	183326236	183962825	3	Hhipl2, Mia3, Taf1a
chr11	17085798	17235200	3	Pno1, Ppp3r1, Wdr92
chr11	20281075	20742270	3	Aftph, Sertad2, Slc1a4
chr11	21164683	21571934	3	Mdh1, Ugp2, Vps54
chr11	51680094	51807621	3	0610009B22Rik, Sar1b, Sec24a
chr11	98551097	98599586	3	Gm12, Ormdl3, Zpbp2
chr12	78748911	78887010	3	Atp6v1d, Eif2s1, Mpp5
chr12	84052151	84109783	3	Acot3, Acot5, Acot6
chr13	49187547	49248706	3	1110007C09Rik, Ninj1, Susd3
chr14	8165991	8259019	3	Acox2, Kctd6, Pdhb
chr15	82329532	82354796	3	Naga, Ndufa6, Smdt1
chr15	98663421	98728198	3	Ccdc65, Fkbp11, Rnd1
chr16	20605458	20621278	3	Alg3, Camk2n2
chr17	33823037	33849774	3	Cd320, Ndufa7, Rps28
chr17	36948355	36965623	3	Ppp1r11, Znrd1, Znrd1as
chr17	46042220	46153551	3	Mad2l1bp, Mrps18a, Rsph9
chr17	56269462	56300286	3	Arrdc5, Plin3, Ticam1
chr17	74299474	74424229	3	Dpy30, Slc30a6, Spast
chr18	74890542	75009933	3	BC031181, Lipg, Rpl17
chr19	6057888	6067867	3	Fau, Tm7sf2, Znhit2
chr2	30132760	30178459	3	D2Wsu81e, Endog, Tbc1d13
chr2	110519123	110739537	3	Muc15, Slc5a12
chr3	103799132	103854618	3	Ap4b1, Bcl2l15, Dclre1b
chr3	104542849	104786017	3	Fam19a3, Ppm1j, Slc16a1
chr3	107230614	107284081	3	A630076J17Rik, Lamtor5, Prok1
chr4	118893085	118934840	3	Olfr1328, Olfr1329, Olfr1330
chr4	135975602	136053371	3	Pithd1, Rpl11, Tceb3
chr5	5580982	6710028	3	Cfap69, Steap1, Steap2
chr5	38220482	38304217	3	Lyar, Otop1, Tmem128

chr5	108797193	109006436	3	Vmn2r10, Vmn2r8, Vmn2r9
chr6	83770414	83838419	3	Nagk, Paip2b, Tex261
chr7	30232074	30289935	3	Ovol3, Polr2i, Wdr62
chr7	30727701	30781066	3	Dmkn, Sbsn, Tmem147
chr7	78895927	79115099	3	Acan, Aen, Isg20
chr7	118777541	118995211	3	Gprc5b, Iqck, Knop1
chr7	127885444	127909664	3	Bckdk, Prss53, Vkorc1
chr7	141471860	141550725	3	Chid1, Polr2l, Tspan4
chr8	3587450	3624235	3	Camsap3, Pet100, Xab2
chr8	35465265	35849466	3	Cldn23, Eri1, Mfhas1
chr8	80922752	82741975	3	Il15, Inpp4b, Usp38
chr8	85080963	85123095	3	Man2b1, Wdr83os, Zfp791
chr8	94819818	94864240	3	Ciapin1, Coq9, Polr2c
chr8	122433596	122476064	3	Mvd, Rnf166, Snai3
chr9	22072246	22117169	3	Cnn1, Ecsit, Elof1
chr9	45906569	45964991	3	Pcsk7, Sidt2, Tagln
chr9	86571988	86757506	3	Me1, Prss35, Rwdd2a
chr9	103188033	103288297	3	1300017J02Rik, Srprb, Trf
chr1	40465504	40768885	4	Il18r1, Il18rap, Slc9a2, Slc9a4
chr1	167204544	167393797	4	Aldh9a1, Mgst3, Tmco1, Uck2
chr11	30976707	31935635	4	Bod1, Chac2, Cpeb4, Stc2
chr19	10064164	10207824	4	Fads1, Fads2, Fen1, Tmem258
chr19	41829970	41918665	4	Frat1, Frat2, Pgam1, Rrp12
chr2	34826243	34982735	4	Cutal, Phf19, Psmd5, Traf1
chr4	129576815	129614257	4	Dcdc2b, Eif3i, Fam167b
chrX	36795597	36902899	4	C330007P06Rik, Nkrf, Slc25a5, Ube2a
chr1	75135215	75192010	5	Abcb6, Atg9a, Cnppd1, Fam134a, Zfand2b
chr1	130670202	130852249	5	AA986860, Fcamr, Pfkfb2, Pigr, Yod1
chr3	89228273	89280951	6	Dpm3, Efna1, Krtcap2, Muc1, Slc50a1, Trim46
chr5	87654061	87943743	7	Csn1s1, Csn1s2a, Csn1s2b, Csn2, Csn3, Odam, Prr27

Supplementary Table 2. DNA binding motifs for STAT5, GR and NFIB in regulatory elements of casein locus.

		TF binding motifs		
		STAT5	GR	NFIB
Csn1s1	E1		x	
	E2			x
	P	x		
Csn2	P	x		
	E1	x		x
	E2	x		
	E3			x
Csn1s2a	E1	x		
	E2	x		
	P	x		
Csn1s2b	E1	x		x
	P	x		
	E2	x		
SE	E1			x
	E2		x	x
	E3	x		
	E4			
Csn3	E1			x
	E2	x	x	x
	P			x

Supplementary Table 3.

Sequences of sgRNA for CRISPR/Cas9 targeted mice.

Target site		sgRNA sequences
SE	E1	5′-AACAACCTAACTCTTGAAATTGG-3′
	E2	5′-TATACAGGAGGTTTGGAACTCCAGG-3′<br>5′-GGAATAATTTGGGGTCAGTGAGG-3′
	E1-4	5′-CGCTAAGCATGTAGGGTCTCAGG-3′<br>5′-TGGGTGTTCTTCCACTAGACAGG-3′
Csn3	E1	5′-GGCTATCCATTTTCTGCTGTAGG-3′<br>5′-TTGTCCAACAGAGATTTTGTTGG-3′<br>5′-AAGTGAAGGTCAGCGTAATGAGG-3′
	E2	5′-CAGGTCATCCTATTGCCTCAAGG-3′<br>5′-GTGTAATGGTTCCCAGAAACAGG-3′
Csn1s1	E1	5′-AATTGATTCAGAACATGTCCAGG-3′
	E2	5′-TGAAAGGATGGCACTCTCTGCGG-3′<br>5′-TTTGCCATGATCTGACTACGTGG-3′
Odam		5′-GTCAGTTAAACTCCAGGTAGGGG-3′<br>5′-ACTTACCGGGGCTGATGATGAGG-3′<br>5′-CCAGTGTTGCTTCTCGTAGAAGG-3′<br>5′-AACTACTTAGTAGGTAGGCCAGG-3′
Csn2	E1	5′-AGATGGTTCACAAACTCAACAGG-3′<br>5′-AGTGAACCCTAATGTAACAGTGG-3′
	E2/3	5′-GCTGTTCTTAATTCAGGCCCAGG-3′<br>5′-GAACTGGCCCTTATCATTACTGG-3′
	P	5′-TCAATTCCAAGAAGTCTACGTGA-3′<br>5′-TTCTTGGGAAAGACAATAGA-3′

Supplementary Table 4.

Characterization of mutant mice generated by CRISPR/Cas9. The GAS, NFIB and GR motifs are highlighted in yellow, blue and pink, respectively.

Target sequence at SE-△E1
ACAACAACCT~~AACTCTTGAAATTGGCAAGCTATCTAAATAACTGGACAACTGCAGTTCAGTTT~~<br>~~TGCCTCTGGGTACACAGGTGGCTGGCCAGTGGCCAGTCCTTTTCAGCTCTGGCTGGCAATGTCA~~<br>~~TTGTCCTGAGGGAATTCAGGAAGGCAGTGGCTTCGGTTGGGTTAGATGGACTTCTCACTTCCCA~~<br>~~TTCTGACCTTGTTTGCCTTGGCTGTTCTCTCATTGGCAGACAAGGTGGCCTGTTCTCATCTGTG~~<br>~~AATAGCTTTATTGATTTCTCTTGTCACATCTAATTTCCTTGTACTAGACTTAATCCATGGAATA~~<br>~~CATCTTGGGTGAATCTGCTCTGTGTTGTTCTCAAATAACATATCAGTCTAAGCCCTTCACAGTG~~<br>~~GAAATCCTGCAGAAGCTGGGTATATGAGTATTTATTTGCACAGCTCTTGAGGGATTATAGCACA~~<br>~~TGCTCTGAGTT~~ATACTAAATT~~CTAAGCCCTTCACAGTGGAAATCCTGCAGAAGCTGGGTATATG~~<br>~~AGTATTTATTTGCACAGCTCTTGAGGGATTATAGCACATGCTCTGAGTT~~ATACTAAATT

Target sequence at SE-△E2
TTATACAGGA~~GGTTTGGAACTCCAGGTTTTTCTGAACTTGTACTTCCATTTTTGACAGCTTTAC~~<br>~~CCAGGTGTTGATGGTGATCTTATTTCCACAAACCTCAACCATCCCAGTCACTTAGACATGATTG~~<br>~~CTCCATTATGGAAAATACACCCTTGAATTATTTCAGTTTGACCTTTAAAGGAAAGAACAAGACA~~<br>~~TTTAGTTTTTCAGAATTCTCCACTGAGAGTTTCTCAGTGTCACTAAGGAGCTGCCAGAGATTTC~~<br>~~TCAAGCACATCCTGCTAAAAGAACAGGATTAGTTCCAGGGTAGATCTTAACAATTCTTCCGCTT~~<br>~~TGGACTCAACCTGTATCCATCAACCTGTGCCAAGAGGAAATATCGGAAAGCTCATCTAATCTTT~~<br>~~AGGTCTAAATGCCATTTTTCCAAGGTACCCATAAAATATACAATGAGAAAATGTACTTCTATAT~~<br>~~TTCAATTCAAATGCCCTTGTAAAAGTGTATTTTACATGCATGCATTAAAAGTTCTTTTAAATAT~~<br>~~CTTTTGTTACAATAATAAAGATTTTGTATATATTTAACTTTGTGTGATTTTCTGGACTTAGAAT~~<br>~~TTTTCATAAAAATAATGGGCATTCTTTGCTGGAAAGTTAACATAACCAACCAGTGAATTTTGTG~~<br>~~TTCCATAGAACTCAAGATGAGTAATTGCTATAAAAGTACACCAGTGGTCAGGCTCCTCCTGCCA~~<br>~~TTCTTGAAACCAAACATCAACCTCACTGA~~CCCCAAATTA

Target sequence at △E2 of SE-△E1/2
TTATACAGGAGGTTTGGAACT~~CCAGGTTTTTCTGAACTTGTACTTCCATTTTTGACAGCTTTAC~~<br>~~CCAGGTGTTGATGGTGATCTTATTTCCACAAACCTCAACCATCCCAGTCACTTAGACATGATTG~~<br>~~CTCCATTATGGAAAATACACCCTTGAATTATTTCAGTTTGACCTTTAAAGGAAAGAACAAGACA~~<br>~~TTTAGTTTTTCAGAATTCTCCACTGAGAGTTTCTCAGTGTCACTAAGGAGCTGCCAGAGATTTC~~<br>~~TCAAGCACATCCTGCTAAAAGAACAGGATTAGTTCCAGGGTAGATCTTAACAATTCTTCCGCTT~~<br>~~TGGACTCAACCTGTATCCATCAACCTGTGCCAAGAGGAAATATCGGAAAGCTCATCTAATCTTT~~<br>~~AGGTCTAAATGCCATTTTTCCAAGGTACCCATAAAATATACAATGAGAAAATGTACTTCTATAT~~<br>~~TTCAATTCAAATGCCCTTGTAAAAGTGTATTTTACATGCATGCATTAAAAGTTCTTTTAAATAT~~<br>~~CTTTTGTTACAATAATAAAGATTTTGTATATATTTAACTTTGTGTGATTTTCTGGACTTAGAAT~~<br>~~TTTTCATAAAAATAATGGGCATTCTTTGCTGGAAAGTTAACATAACCAACCAGTGAATTTTGTG~~<br>~~TTCCATAGAACTCAAGATGAGTAATTGCTATAAAAGTACACCAGTGGTCAGGCTCCTCCTGCCA~~<br>~~TTCTTGAAACCAAACATCAACCTCACTGA~~CCCCAAATTA

Target sequence at SE-△E4
AGTATAGTCA~~TAAATATAACAGCCAAAACACCCATCTGAAAATATTACAAATCACAAGAATTCC~~
~~TTATTAGATAAAAATAGAAATTGATACTACTTATAAATGAGTCTGCCTAGTAATGTAACAGTTA~~
~~TTCCCGAATCCTGGTGTTTCTGCAACTTTTGGTTCTTTCTAAAATACATTCTAAGAGAAGGAAG~~
~~TTGTCTGTGATTCTGGCTTATTAATTCCTGGCTTCAGTTGCTTCCAGTAATTGTTATTGCAAGT~~
~~AGAAAACCTCCATGGGAAAGGAATAGAGAACTACCATGCTTCTTGGCTAAAATGTGGCCTGAAC~~
~~AATAAGAGCTATTGCTGAATGACCTTCATGCTTAGTGATTTCAACTAAATACACTGATGATTGG~~
~~AACATGCCTGGATTTTTTTTTTCCTTCATGGCAGATACAAGCAACTTTAGGCTCTTGAAGAAGC~~
~~AAAAGTACATAGCTTATTCAACAGCTATCACATAGAAAATTCTCTAACAATCTAAGATTTGCCA~~
~~CAGAAAATTCATAAGCTGAAAGGTATTGACTGCAAGAGTCTATATGGAGATTATAGAGTGCAA~~
~~GAGATCTGGAGAATTTTCAACAAATGCTCAAAAGAAATTTTGATCATTCTAAGAGTGTAGCTCT~~
~~TGGTGAGCTCACTCCTGTCTAGTGGAAGAACACCCATCTAAGAATACCTGTGCAGCATGAATTG~~
~~GGCTGTTGGGATTTAGAAATAAGAGGGCACAAAGATGAGTGGGTAGGAAAGGAGCTTGTCTGGC~~
~~GAGAGTTGGGTGATGGATGAATGCGATAAAAACTTCTTGTATGAAACTCTCAAATACTAATAAA~~
~~AAAAGAAATCAACAGAATCAAAAATGCTTGCACTATCCAGTGTTTTGGT~~CCCAAATAAAT

Target sequence at △SE
9kb<br>AACCTCCTGAG--------------------…--------------------TGGAAGAACA

Target sequence at △Odam
9.1kb<br>GAGTGGGAAT--------------------…--------------------AGCAACACTG

Mice	Target sequence at Csn3-△E1
	240bp 173bp
WT	TCCATTTTCTG…GTCATCCAACAAAATCTCTGTT…TGAAGGTCAGCGTAATGAGGCACTTA
ΔE1	TCCATTTTCTG…GTCATC---------------…-------------------CACTTA

Target sequence at Csn3-△E1
GTTCAATATTATG~~ATCTTCATAAAAAAATATCTCAGTCAAAAAAAAAATGTCCTCACTATGACCTC~~
~~CTCCCATAGTACTTTATTTCTGCGCTTTATAAGTCTAACCATTCTTTGGTCTTTGTTCTTCTTG~~
~~AGTTTCATGTGTTTTGCAAATTGTATCTTATATCTTGGGTATTCTAAGTTTCTGGGCTAATATC~~
~~CACTTATAGCTGACACCATTGCATACACTAGCAAGATTTTGCTGAAAGGACCCTGATATAGCTG~~
~~TCTCTTGTGAGGCTATGCTGGGGCCTAGCAAACACAGAAGTGGAGGCTCATAGTCAGCTATGAT~~
~~GGATCACAGGGCCCCCAATTGAGGAGCTAGAGAAAGTACCCAAGGAGCTAAAGGATCTGCAAT~~
~~CCTATAGGTGGAACAACAATATGAACTAACCAGTACCCCCTGGAGCTCGTGTCTCTAGCTGCAT~~
~~ATGTATGAGAAGACGGCCATCAGTGGAAAGAGAGGCCCATTGGTCTTGCAAACTTTATATGCCT~~
~~CAGTACAGGGGAACTCCAGGGCCAAGAAGTGGGAGTGGGTGGGTAGGGGAGTGGGTGGGGGGAG~~
~~GGTATTGTGGACTTTTGGGATAGCATTGGAAATGTAAATGAAGAAAATACCTAATAATAATAAC~~

~~AATAGTAACAATAATAATAATAATAAAATGAGTCTAACCACGCTACAGCTTCTAGTAGAACAGT~~
~~CCATATCATTTTCTGCATCTTACTTAAGCCTTGGAATATGACATCAATCTAAATGTACTCCAGT~~
~~CCTTAGATGCTCAGTGACTGCTAAATTGAATATATTAACAAGAATTAACAATTCCTAATATTTT~~
~~TAAAAAGAGCATTTTCACACATATCAATGTGGAAGGTGGTAAAAGGTTCATTCTCTCAGGAAAT~~
~~GAAATTATTAAAATCAGTATTTAAATGTTGACAAATTTATTCTATTCAATTACAATTTTAGGGC~~
~~AGCAAGTTGAGTAATTTCTTGGCTATCCATTTTCTGCTGTAGGAACTCTAGTCATAATCAAGTC~~
~~ATAAATAAAATTTTGTTCTGAATACTACAATTCATACATATTGTTATACATGAATTAGACATTC~~
~~AAATTTCTGAGGATTTTAATTTATCTAGTGTTGTGTGGGCTTTTTCTAGAGCAAAATTATATAA~~
~~AAAGACAGTTTGTCCTGTTCACTGTCTCTTCCTACTAACCTGATGGTGAACACAGCGTAATGCC~~
~~ATGCAGGAAGGAAAGGTACAGAAGTCATCCAACAAAATCTCTGTTGGACAATGAGCCTTTATTA~~
~~TGTCACAATCGCTGCCCTTGTCTCGGTGGTAAATTATCAGGAAATTAGAAAAGTTCTAAGACTG~~
~~TATCTTTCCACCACAATAAATTTATGCACAGATGAGACCCTTTGCACCAACCATGGAAACATTG~~
~~TTCTCTGTGTGCATCCTGGCTGAAAGTGAAGGTCAGCGTAATGAGGCACTTAGCTATATTGGTG~~
~~GACAGAAACTTCTGTCTTCCTTAGTCATTTGCAGGGATTACAGACTCCTTATGCACTTAGATAA~~
~~TAATCCCGCTAAATACTAGGACATCAGGAGGACCGAGTCTTATGGTAAATTGATTTTATAGGGA~~
~~TCACTTCTGAAACCTATGCCAACATATTGTGTGCCATTGTAGGACTGGAGAAAGACCAGCATGG~~
~~AAGAAAAATGGAGGCCTAGAATCATGGTCAACTAAGTCTCTAGGGGCCTAAGACTAACTAACA~~
~~AAAGCTACTATAAACTAAGAGAATTTTGGGTAATATATTCACCTTTCTTTCTCTGTATCTGTGT~~
~~CTGTGTCTGTGTCTGTGTCTGTGTCTGTGTCTGTGTCTGTGTCTGTGTCTGTGTCTGTGTCTGT~~
~~GTGTCTGTGTCTATCTGTCTCTCTCTCTCTCTGTCTCTCTCTCTCTCTCTCTTATCTTTGAA~~
~~TCTCAGATTAGAGCTTTGATTTTATTCTTTTATTCACTTCCCATTATGACACGGTAACATTTTG~~
~~GGGGGTTAGGGAAGAGATCCTACCATGTACTTTTGATTATCCTATGATGCAAATATATTGGTTA~~
~~AAATTGACCAGTCTGTCCATTAGAAAATTTAAGAAATATCTTTA~~ATCTCTGA

Target sequence at Csn3-△E2-S

CCACGCCAGAAATGCTTTTCTGAAGAGAAGGGGTCCCAGAATTCACTCAGCTATTCTGGACACA
TATTCCCCTTCTGAAATTATATATGTATGTGTATAAATATATATATACACACACACATATATAT
ATATATATTTTTAGATACATTTGAGTCTTTTTTGGTTATTTTTTAATCAAATAATAAAACAGCT
TAAAGCATGTATTCTTAATTGATGATTGAGAAAGGACTGGAATGGAATAAAGCATCTTCAGAGT
CTGATGAACCATAACCAGACCATAAATGTCCACCTTTATTCCTAGGCCCTT~~GAGGCAATAGGAT~~
~~GACCTGACAGTCTCCATACACAGGCTGCTTGTATATTGTGTGTGTCAGAGAAAGAACGAGAGAA~~
~~GCAAACTTCTAAGAAATAGAAAGCCAGGGCTTATTGTGGAGAAGCTGCACAAGTATTCTCAGGA~~
~~TTCAGAACAATGTTCCCTGCTTTCTAAGACGTGTAATGGTTCCCAGAAACAGG~~CAGGAATACAT
AAAAATTATACAATGCTGACATTATTTCTGCTCTGCATTCTGGCTGCTCTGCAGGGATTCCAGT
AAATACCTCTGATGAGGTTTTCACAAAGCAAAGTCATTGTTAGCATTACCCTGGGGTGGGGGGA
GGGACAGGTAAAGCAGGATATCATTTAGGCAAAATACATGTTTGGCTTCATTTTACTAAAATTT
TTACAGCATTGGCAGTTTTGCAATCCATGCGCTAAATCAGAATGATCTGACTCTAAAATAGAAC
AATGATTCCTAACTCAGTTACTTAATATTGGGCATCCAAAATGGGACACTTTAGGGGTTCATTA
AAACAGCATGTGTTCAATGTTTGAACAATTGAGGAATACACGCTGAAGCAAAAACGCAAATGCT
CAGCCTTCACAGCAAAGAAAATGTTTCATGCATGAATTTAACTTACAAAACGCACACATAATTA
AGACTGCTGATTTTTATTTTTAAAATAAGTAGTTGTACATTTGGAAGAACAATGACCAACATGG
ACCATACTAAATATAAAGTCCCTTGAAGACATAGTTCTTCAAGTAAAAACACTTAAAATGTGAA
AAATAATTGTGGCTATTGGGTTTTAATTTTAT

Target sequence at Csn3-△E2-S/N

CCACGCCAGAAATG~~CTTTTCTGAAGAGAAGGGGTCCCAGAATTCACTCAGCTATTCTGGACACA~~
~~TATTCCCCTTCTGAAATTATATATGTATGTGTATAAATATATATATACACACACACATATATAT~~
~~ATATATATTTTTAGATACATTTGAGTCTTTTTTGGTTATTTTTTAATCAAATAATAAAACAGCT~~
~~TAAAGCATGTATTCTTAATTGATGATTGAGAAAGGACTGGAATGGAATAAAGCATCTTCAGAGT~~
~~CTGATGAACCATAACCAGACCATAAATGTCCACCTTTATTCCTAGGCCCTTGAGGCAATAGGAT~~
~~GACCTGACAGTCTCCATACACAGGCTGCTTGTATATTGTGTGTGTCAGAGAAAGAACGAGAGAA~~
~~GCAAACTTCTAAGAAATAGAAAGCCAGGGCTTATTGTGGAGAAGCTGCACAAGTATTCTCAGGA~~
~~TTCAGAACAATGTTCCCTGCTTTCTAAGACGTGTAATGGTTCCCAGAAACAGGCAGGAATACAT~~
~~AAAAATTATACAATGCTGACATTATTTCTGCTCTGCATTCTGGCTGCTCTGCAGGGATTCCAGT~~
~~AAATACCTCTGATGAGGTTTTCACAAAGCAAAGTCATTGTTAGCATTACCCTGGGGTGGGGGA~~
~~GGGACAGGTAAAGCAGGATATCATTTAGGCAAAATACATGTTTGGCTTCATTTTACTAAAATTT~~
~~TTACAGCATTGGCAGTTTTGCAATCCATGCGCTAAATCAGAATGATCTGACTCTAAAATAGAAC~~
~~AATGATTCCTAACTCAGTTACTTAATATTGGGCATCCAAAATGGGACACTTTAGGGGTTCATTA~~
~~AAACAGCATGTGTTCAATGTTTGAACAATTGAGGAATACACGCTGAAGCAAAAACGCAAATGCT~~
~~CAGCCTTCACAGCAAAGAAAATGTTTCATGCATGAATTTAACTTACAAAACGCACACATAATTA~~
~~AGACTGCTGATTTTTATTTTTAAAATAAGTAGTTGTACATTTGGAAGAACAATGACCAACATGG~~
~~ACCATACTAAATATAAAGTCCCTTGAAGACATAGTTCTTCAAGTAAAAACACTTAAAATG~~TGAA
AAATAATTGTGGCTATTGGGTTTTAATTTTAT

Target sequence at Csn1s1-△E1

TACTAGCACTGAACACTATGTATCATGGAAATTTTCCTTGTAAAGAAGTTCTTGGTAGACTCAA
AATAATTGCAATTGAT~~TCAGAACATGT~~CCAGGAAGTACACAATGTTGATTTGGAAGACTTTATC
TGTGATATCTCAGTTGCTTAGTGTGTCACTTAGTACTTTATTATCTCAAAATTATGGATAACAA

Target sequence at Csn1s1-△E2

CTACACGTGCTGGACAAGCCTGTGATCATAAAGATTTCAAACAAGAGCTGAAGGCTGAGGTCAG
AAGAGGTGAAGTCCACCCGGAGGCCCGCAGAGA~~GTGCCATCCTTTCAGGCTTCCTCCTTCCTCC~~
~~TGAATAAGGAGAATTTCCAACCTATGAGACAAGAACTTTGACACTCCACATTCTTCCGTGTTCT~~
~~GTTCCTGTGCCTTACTTTCCAACATCACCACCCTTTGTTCTCTGTCTTGGCACTGTAATCTTTG~~
~~CAAACCTTTGCCATGATCTGA~~CTACGTGGTACATTCCTCTCCGATCCTGTAGCTCATACCCTTC
TGCTCAATCTTTATGAAGTCATTTGCAATTTCTTCATGTTAGAGTAATTTTTCCCTTTCAGCAT

Target sequence at Csn2-△E1

AAACATTAAAATATTTTGATCTTA~~GAATAGTGATAATTGAAATATTTTCTGCTTTCATAAATTT~~
~~AAATTAGAATTGTATCAGAGGAATAAAATGATTCTTATTTCATGTAGCATCATTTTAAAATGTG~~
~~TTTACAAAACTTGTTCGTGGTATACAGTGTAAATAAGTATAAATAAAGAAAAAAGTTAACTGGT~~
~~TAATTTTGTATTAAACTTAACTTATTCTTATTCATTATTCTTAATTAATGAGGCTTTGACTAG~~
~~TGAATATTTTTTTATTATCAATTGCAGAAAAATAAATATATTTTCATTTTTCCTGTTGAGTTTGT~~
~~GAACCATCTTTTACTAACCACAATTCTGTATTCAGAATTTAATGACAAAGACTAAGTTCCAGTC~~
~~AAATTAACTTTAATTTTGTGTACATTCTAAGTCAGAGAGAATCTAGAAAAGGAAATATTGAAAA~~
~~ATTTCCATTGCCTTAAGCCAATTTAGGAAACAGTTTGGCTGCCCTGTGATTGCCAATGCCAGCC~~
~~AACAATATATGACTTCTAAGAAAATGAATACAGAAGTGAGCAAGCCTTATCCATGAAACATTTC~~
~~CTATTAATTTCTCAGCAAAATGCTTTACATCTTGTGCAATAGATCCAGTATTTCCTTGAGACTC~~

~~TCTCACTAACATTAAGCTGTTTTAATCAATATACTTATAGATTGGTCTGAGCCAGAAAATGAGA~~
~~AAATTGTGATCAAGGAAAGAGTGAATAATATACTAGAGTTAGAGAATATTCCAGTGAAAAGTAG~~
~~ACAAACTACAAGCTTAAAATAAAGTTGATGTCTTCAGTAAGGTAAACTTTAGACCACAATTATA~~
~~TAAGAGGCAAAGTATAGAAACCTAGGCTTGCTCAACTTAAGATCTGCAGTTTTCACAAGGAAAG~~
~~AAACATCCTCACTGAGATTTCTCAATAGCCTTGGAACAAACTAGATACAGTCAGTGCAATGTGT~~
~~CCTAGGGCTTGGGTAATGGGCCTGCAATAGACAAACATGAGATTTGAGTGAACCCTAATGTAAC~~
~~AGTGGAGGTCATATAAATGCATAGAAATAGAATTGCTTTGTTTAATTTGTTGCAGCACAGAGAG~~
~~TAAAGTAGACTACCACTCAAAAAGACGAAGATTAGAAGAGACCCTAGATATCATTTGTAAAATT~~
~~TAGACTTGGAAAATCGTGTTATTTAAGATAATATAGCTATTTAGAAATATAAGCAGAACTAGCT~~
~~AATATTCAAAACACTCTTTAGCACAACACCAATGTAGGGAAAGAAAGTAAACAGTGCAATCAAT~~
~~GTAATATTTCAAGTGTAAATAATATAATTAACTAAAAATGCATGTAGAAAGGAATAGCTAATAG~~
~~CATTTCATATGTAAGTGGATATTTTAATTCAATCCATGAGATTATTTTAAGTATAATAAA~~ATG
GCGTATTACTTAAAATATTGTTGTTAAATTGATCTATAAATATAAGCAAAAGTCATACAATTAA
AAAAATATTGCCCATAAAGCCTAACTAATACAATGTATTTTTCATCAGGTACTAATTTTTTTGT
TTACACGGAATTATAAATTTTCATTTGTGTTCAGGAGAGGTTTATGAAGCACAGATAGCCCGAA
TGTT

Target sequence at Csn2-△E2/3

TCCCTCAACTTATGATCTTGAAAATGTTTACTGACACCAAGAGAAAGAGAAATGTCCCCTAAAT
AAGGATTGATTATTTAGAGTAGACCGTGGTGATATA~~AGGGAAGGCTCAAATCACACATTCATAT~~
~~GAAATGCTGAAGCCAGGAAATGATAAAGGCACACAAACTCTTGAGAAGGAGTTCTGAAAATCAA~~
~~CCCAGTGCTTTGCAATCTTTCCAAGGCTTGATTATGATGAGGAAATGTGGACATGCACCACGGA~~
~~GTTTGGGAAACAACACTAGAAATGAACAGAGTTCAAAGAAGGCAGGAAAGAGACAATGGTGGCA~~
~~CCAGCCAGGGTAGCATGCCTGTATAATCCATAGGGCATTGTTAGGATTCTTAGAAAAGTATTGC~~
~~TTCATTAATGGACTGAATTTCACTCTAAACAAAATGGTTCCCTGTTCCTCATTGCTTAATAAGG~~
~~CTGGAAGCTAGACT~~..9kb..
....~~TGTCCCTAGTACTAGGAAATGCTGCAAGCACACTGCCCAGGAATCACAGT~~
~~ATAGCTGGCTCTGGTTGCAGGAATTGCAGATGAGTCACCCAAAGGGTGTGAACAAGGAAGGGCT~~
~~GGCCCTGCCACACTCATCTCCCATATAGGTGCCATGGATGAAGGAGAGATAGTCTCATCTCTAC~~
~~TTGCTACTTGTGCCAAAAGAGAGACATGGCCCTGGCAGTCATGAGAGTAGGAAATCGTACATTA~~
~~ACCCTTACCAGTTTCATCACTCAGGAGAGATGGCCCTGTATTTCATCTGGACAACATGCTAGAG~~
~~CTGGCCCTACATCAGGTTAGCTGGCACTGGATATGTCTGTGTGAGAGCTAGCCCTGCCTCTTGT~~
~~CCACCTTGTGATTGTGAGGATGAGGGAGAGATGCCCTCCTCTCCTCCCCATTCTTTTGTCATCT~~
~~GTATCATGTGGGAGACCTGTCCCTGGGGTCATAATATTAGAAGAACTGGCCCTTATCAT~~TACTG
GGTGCAACACTCAGGCTCTGTACCTCATCTGTGCAATACAGTAGGGTTAGCC

Mice	Target sequence at Csn2-P
WT	TTCCAAGAAGTCTACGTGATTAGAAAATGGTTTCTTTCTATTGTCTTTCCCAAGAA
ΔP	TTTCAAGAAGTCTACGTGATTAGAAAATGGTTTCTTTCTATTGTCTTTCCCAAAAA
ΔP-A	TTTCAAGAAGTCTACGTGATTAGAAAATGGTTTCTTTCTATTGTCTTTCCCAAGAA
ΔP-B	TTCCAAGAAGTCTACGTGATTAGAAAATGGTTTCTTTCTATTGTCTTTCCCAAAAA
