# Supplementary material for: Cell-specific and shared regulatory elements control a multigene locus active in mammary and salivary glands
Source: Nat Commun. 2023 Aug 17;14:4992. doi: 10.1038/s41467-023-40712-0 (PMC10435465; doi:10.1038/s41467-023-40712-0)
Supplement: Supplementary file 3 — Description of Additional Supplementary Files [file 41467_2023_40712_MOESM3_ESM.pdf]

Supplementary Data

Supplementary Data 1. (separate Excel file)

List of all genes with normalized read counts in each replicate at virgin, p6, L1 and L10 mammary tissue from WT mice, log_2 (fold change), their *p*-value and adjusted *p*-value. *P*-values were calculated using a paired, two-side Wilcoxon test and adjusted *p*-value (pAdj) corrected using the Benjamini–Hochberg method.

Supplementary Data 2. (separate Excel file)

List of all genes with normalized read counts in each replicate at p18 mammary tissue from WT and ΔSE mutant, log_2 (fold change), their *p*-value and adjusted *p*-value. *P*-values were calculated using a paired, two-side Wilcoxon test and adjusted *p*-value (pAdj) corrected using the Benjamini–Hochberg method.

Supplementary Data 3. (separate Excel file)

List of all genes with normalized read counts in each replicate at p6 mammary tissue from WT and ΔSE mutant, log_2 (fold change), their *p*-value and adjusted *p*-value. *P*-values were calculated using a paired, two-side Wilcoxon test and adjusted *p*-value (pAdj) corrected using the Benjamini–Hochberg method.

Supplementary Data 4. (separate Excel file)

List of all genes with normalized read counts in each replicate of salivary tissue from WT and ΔSE mutant, log_2 (fold change), their *p*-value and adjusted *p*-value. *P*-values were calculated using a paired, two-side Wilcoxon test and adjusted *p*-value (pAdj) corrected using the Benjamini–Hochberg method.

Supplementary Data 5. (separate Excel file)

List of all genes with normalized read counts in each replicate of salivary tissue from WT and ΔOdam mutant, log_2 (fold change), their *p*-value and adjusted *p*-value. *P*-values were calculated using a paired, two-side Wilcoxon test and adjusted *p*-value (pAdj) corrected using the Benjamini–Hochberg method.

Supplementary Data 6. (separate Excel file)

List of all genes with normalized read counts in each replicate at p18 mammary tissue from WT and ΔCsn3-E2-S/N mutant, log_2 (fold change), their *p*-value and adjusted *p*-value. *P*-values were calculated using a paired, two-side Wilcoxon test and adjusted *p*-value (pAdj) corrected using the Benjamini–Hochberg method.

Supplementary Data 7. (separate Excel file)

List of all genes with normalized read counts in each replicate at L1 mammary tissue between WT and Csn2-ΔE1/2/3, Csn2-ΔP or Csn2-P-E1/2/3 mutants, log_2 (fold change), their *p*-value and adjusted *p*-value. *P*-values were calculated using a paired, two-side Wilcoxon test and adjusted *p*-value (pAdj) corrected using the Benjamini–Hochberg method.

Supplementary Data 8. (separate Excel file)

List of all genes with normalized read counts in each replicate at L10 mammary tissue between WT and Csn2-ΔE1/2/3, Csn2-ΔP or Csn2-P-E1/2/3 mutants, log_2 (fold change), their *p*-value and adjusted *p*-value. *P*-values were calculated using a paired, two-side Wilcoxon test and adjusted *p*-value (pAdj) corrected using the Benjamini–Hochberg method.

Supplementary Data 9. (separate Excel file)

List of all genes with normalized read counts in each replicate at virgin mammary tissue between WT and Csn2-ΔP or Csn2-P-E1/2/3 mutants, log_2 (fold change), their *p*-value and adjusted *p*-value. *P*-values were calculated using a paired, two-side Wilcoxon test and adjusted *p*-value (pAdj) corrected using the Benjamini–Hochberg method.

Supplementary Data 10. (separate Excel file)

List of GEO data set used in this study and harvested tissues. Spearman correlation score between ChIP-seq replicates.
