# Supplementary material for: Cell-specific and shared regulatory elements control a multigene locus active in mammary and salivary glands
Source: Nat Commun. 2023 Aug 17;14:4992. doi: 10.1038/s41467-023-40712-0 (PMC10435465; doi:10.1038/s41467-023-40712-0)
Supplement: Supplementary file 14 — Reporting Summary [file 41467_2023_40712_MOESM14_ESM.pdf]

nature portfolio

Corresponding author(s): Lothar Hennighausen

Last updated by author(s): Jul 27, 2023

Reporting Summary

Nature Portfolio wishes to improve the reproducibility of the work that we publish. This form provides structure for consistency and transparency in reporting. For further information on Nature Portfolio policies, see our Editorial Policies and the Editorial Policy Checklist.

Statistics

For all statistical analyses, confirm that the following items are present in the figure legend, table legend, main text, or Methods section.

n/a	Confirmed	
☐	☒	The exact sample size (*n*) for each experimental group/condition, given as a discrete number and unit of measurement
☐	☒	A statement on whether measurements were taken from distinct samples or whether the same sample was measured repeatedly
☐	☒	The statistical test(s) used AND whether they are one- or two-sided<br>*Only common tests should be described solely by name; describe more complex techniques in the Methods section.*
☒	☐	A description of all covariates tested
☐	☒	A description of any assumptions or corrections, such as tests of normality and adjustment for multiple comparisons
☐	☒	A full description of the statistical parameters including central tendency (e.g. means) or other basic estimates (e.g. regression coefficient) AND variation (e.g. standard deviation) or associated estimates of uncertainty (e.g. confidence intervals)
☐	☒	For null hypothesis testing, the test statistic (e.g. *F*, *t*, *r*) with confidence intervals, effect sizes, degrees of freedom and *P* value noted<br>*Give P values as exact values whenever suitable.*
☒	☐	For Bayesian analysis, information on the choice of priors and Markov chain Monte Carlo settings
☒	☐	For hierarchical and complex designs, identification of the appropriate level for tests and full reporting of outcomes
☐	☒	Estimates of effect sizes (e.g. Cohen's *d*, Pearson's *r*), indicating how they were calculated

Our web collection on statistics for biologists contains articles on many of the points above.

Software and code

Policy information about availability of computer code

Data collection	ChIP-seq and RNA-seq data in GEO were downloaded using Sratoolkit (version 2.10.9) and newly generated ChIP-seq and RNA-seq reads were collected using HCS 3.4.0 software for HiSeq 3000 and NovaSeq control software v1.7.5 for NovaSeq 6000.
Data analysis	FastQC tool (version 0.11.9); Trimmomatic (version 0.36);Bowtie (version 1.2.2); Samtools (version 1.8 and 1.17); Picard (version 2.9.2 and 2.27.3); Homer (version 4.9.1); DeepTools (version 3.1.3); IGV(version 2.5.3); MACS (version 2.2.7.1); Bedtools (version 2.29.2); STAR RNA-seq (version 2.5.4a); HTSeq (version 0.9.1) ; R (version 3.6.3); Bioconductor (version 3.10); DESeq2; RUVSeq package; dplyr; ggplot2;GraphPad Prism 9 (version 9.5.1); TrimGalore (version 0.6.7); Bismark (version 0.23.0)

For manuscripts utilizing custom algorithms or software that are central to the research but not yet described in published literature, software must be made available to editors and reviewers. We strongly encourage code deposition in a community repository (e.g. GitHub). See the Nature Portfolio guidelines for submitting code & software for further information.

Data

Policy information about availability of data

All manuscripts must include a data availability statement. This statement should provide the following information, where applicable:

- Accession codes, unique identifiers, or web links for publicly available datasets
- A description of any restrictions on data availability
- For clinical datasets or third party data, please ensure that the statement adheres to our policy

All data were obtained or uploaded to Gene Expression Omnibus (GEO). ChIP-seq, RNA-seq, and Bisulfite-seq data of wild-type and mutant tissues are under

GSE25105, GSE67386, GSE74826, GSE115370, GSE127144, GSE161620 and GSE231441. Data set used in the study was listed in Supplementary Table 14.

Research involving human participants, their data, or biological material

Policy information about studies with human participants or human data. See also policy information about sex, gender (identity/presentation), and sexual orientation and race, ethnicity and racism.

Reporting on sex and gender	not applicable
Reporting on race, ethnicity, or other socially relevant groupings	not applicable
Population characteristics	not applicable
Recruitment	not applicable
Ethics oversight	not applicable

Note that full information on the approval of the study protocol must also be provided in the manuscript.

Field-specific reporting

Please select the one below that is the best fit for your research. If you are not sure, read the appropriate sections before making your selection.

☒ Life sciences ☐ Behavioural & social sciences ☐ Ecological, evolutionary & environmental sciences

For a reference copy of the document with all sections, see nature.com/documents/nr-reporting-summary-flat.pdf

Life sciences study design

All studies must disclose on these points even when the disclosure is negative.

Sample size	15 mutant mouse lines with deletions of regulatory elements were generated. No statistical methods were used to determine sample size. In general, at least three independent replicates were performed in all experiments. When possible, we have aimed for the replication of the animal experiments in at least two different cohorts. The sample size used for each experiment is indicated at the corresponding figure legend in the manuscript.
Data exclusions	None of data were excluded in the data analysis.
Replication	The number of independent replicates for each experiment is indicated at the corresponding figure legend in the manuscript. In general, at least three independent replicates and two independent ChIP-seq replicates were performed.
Randomization	In all animal studies, groups were allocated randomly. Age and gender-matched animals were used in all the experiments.
Blinding	For all animal studies, the investigators were blind to group allocation. Blinding was not applicable to the rest of experiments.

Reporting for specific materials, systems and methods

We require information from authors about some types of materials, experimental systems and methods used in many studies. Here, indicate whether each material, system or method listed is relevant to your study. If you are not sure if a list item applies to your research, read the appropriate section before selecting a response.

Materials & experimental systems

n/a	Involved in the study	
☐	☒	Antibodies
☒	☐	Eukaryotic cell lines
☒	☐	Palaeontology and archaeology
☐	☒	Animals and other organisms
☒	☐	Clinical data
☒	☐	Dual use research of concern
☒	☐	Plants

Methods

n/a	Involved in the study	
☐	☒	ChIP-seq
☒	☐	Flow cytometry
☒	☐	MRI-based neuroimaging

Antibodies

Antibodies used	5-10 ug of antibodies were added in 1 mg of total proteins (1ml solution).

STAT5A (Santa Cruz Biotechnology, sc-1081 and sc-271542), GR (Thermo Fisher Scientific, PA1-511A), NFIB (Sigma-Aldrich, HPA003956), MED1 (Bethyl Laboratory, A300-793A), H3K27ac (Abcam, ab4729), RNA polymerase II (Abcam, ab5408), H3K4me1 (Active Motif, 39297) and H3K4me3 (Millipore, 07-473)

Validation

Previous papers from our group - PMID: 27376239, 30285185, 27694626, 32636391, 28009300, 28714474, 28334928, 32321991, 26446995, 27215382, 127139

STAT5A - PMID: 27376239, 30285185, 27694626, 32636391, 28009300, 28334928, 32321991, 26446995, 127139
GR - PMID: 27376239, 30285185, 27694626, 32636391, 28009300, 28334928, 127139
NFIB - PMID: 27376239
MED1 - PMID: 27376239, 30285185, 26446995
H3K27ac - PMID: 27376239, 30285185, 27694626, 32636391, 28009300, 28714474, 28334928, 32321991, 26446995, 127139
RNA polymerase II - PMID: 27376239, 30285185, 32636391, 28334928, 32321991, 26446995, 127139
H3K4me1 - PMID: 27215382, 127139
H3K4me3 - PMID: 27376239, 32636391, 28009300, 28714474, 32321991, 26446995, 27215382, 127139

Animals and other research organisms

Policy information about studies involving animals; ARRIVE guidelines recommended for reporting animal research, and Sex and Gender in Research

Laboratory animals

C57BL/6N mice (Charles River Laboratories, MD) were used to generate CRISPR/Cas9 targeted mice and as a normal condition. All mice were 8-12 weeks and the mammary gland tissues at specific time points were harvested from pregnant or lactating females. Mice were housed in an environmentally controlled room (22-24 °C, with 50 ± 5% humidity and 12 h / 12 h light–dark cycle).

Wild animals

No wild animals were used in the study.

Reporting on sex

Mammary gland tissues were collected from females mice and salivary gland tissues were collected from male mice.

Field-collected samples

Mammary gland tissues from specific stages during pregnancy and lactation were harvested, and stored at -80°C until being used in experiments.

Ethics oversight

All animals were housed and handled according to the guidelines of the Animal Care and Use Committee (ACUC) of the NIH (https://oacu.oir.nih.gov) and all animal experiments were approved by the ACUC of National Institute of Diabetes and Digestive and Kidney Diseases (NIDDK, MD) and performed under the NIDDK animal protocol K089-LGP-17.

Note that full information on the approval of the study protocol must also be provided in the manuscript.

Plants

Seed stocks

Report on the source of all seed stocks or other plant material used. If applicable, state the seed stock centre and catalogue number. If plant specimens were collected from the field, describe the collection location, date and sampling procedures.

Novel plant genotypes

Describe the methods by which all novel plant genotypes were produced. This includes those generated by transgenic approaches, gene editing, chemical/radiation-based mutagenesis and hybridization. For transgenic lines, describe the transformation method, the number of independent lines analyzed and the generation upon which experiments were performed. For gene-edited lines, describe the editor used, the endogenous sequence targeted for editing, the targeting guide RNA sequence (if applicable) and how the editor was applied.

Authentication

Describe any authentication procedures for each seed stock used or novel genotype generated. Describe any experiments used to assess the effect of a mutation and, where applicable, how potential secondary effects (e.g. second site T-DNA insertions, mosiacism, off-target gene editing) were examined.

ChIP-seq

Data deposition

☒ Confirm that both raw and final processed data have been deposited in a public database such as GEO.

☒ Confirm that you have deposited or provided access to graph files (e.g. BED files) for the called peaks.

Data access links
May remain private before publication.

https://www.ncbi.nlm.nih.gov/geo/query/acc.cgi?acc=GSE231441

Files in database submission

delCsn-SE_SG_H3K27ac_rep1
delCsn-SE_SG_H3K27ac_rep2
delCsn-SE_SG_PolII_rep1
delCsn-SE_SG_PolII_rep2
delCsn1s1-E1_L1_GR_rep1
delCsn1s1-E1_L1_GR_rep2
delCsn1s1-E1_L1_H3K27ac_rep1

delCsn1s1-E1_L1_H3K27ac_rep2
delCsn1s1-E1_L1_NFIB_rep1
delCsn1s1-E1_L1_NFIB_rep2
delCsn1s1-E1_L1_PolII_rep1
delCsn1s1-E1_L1_PolII_rep2
delCsn1s1-E1_L1_STAT5_rep1
delCsn1s1-E1_L1_STAT5_rep2
delCsn1s1-E2_L1_GR_rep1
delCsn1s1-E2_L1_GR_rep2
delCsn1s1-E2_L1_H3K27ac_rep1
delCsn1s1-E2_L1_H3K27ac_rep2
delCsn1s1-E2_L1_NFIB_rep1
delCsn1s1-E2_L1_NFIB_rep2
delCsn1s1-E2_L1_PolII_rep1
delCsn1s1-E2_L1_PolII_rep2
delCsn1s1-E2_L1_STAT5_rep1
delCsn1s1-E2_L1_STAT5_rep2
delCsn2-E1_L1_GR_rep1
delCsn2-E1_L1_GR_rep2
delCsn2-E1_L1_H3K27ac_rep1
delCsn2-E1_L1_H3K27ac_rep2
delCsn2-E1_L1_PolII_rep1
delCsn2-E1_L1_PolII_rep2
delCsn2-E1_L1_STAT5_rep1
delCsn2-E1_L1_STAT5_rep2
delCsn2-E23_L1_GR_rep1
delCsn2-E23_L1_GR_rep2
delCsn2-E23_L1_H3K27ac_rep1
delCsn2-E23_L1_H3K27ac_rep2
delCsn2-E23_L1_PolII_rep1
delCsn2-E23_L1_PolII_rep2
delCsn2-E23_L1_STAT5_rep1
delCsn2-E23_L1_STAT5_rep2
delCsn2-E123_L1_GR_rep1
delCsn2-E123_L1_GR_rep2
delCsn2-E123_L1_H3K27ac_rep1
delCsn2-E123_L1_H3K27ac_rep2
delCsn2-E123_L1_PolII_rep1
delCsn2-E123_L1_PolII_rep2
delCsn2-E123_L1_STAT5_rep1
delCsn2-E123_L1_STAT5_rep2
delCsn2-P_L1_GR_rep1
delCsn2-P_L1_GR_rep2
delCsn2-P_L1_H3K27ac_rep1
delCsn2-P_L1_H3K27ac_rep2
delCsn2-P_L1_PolII_rep1
delCsn2-P_L1_PolII_rep2
delCsn2-P_L1_STAT5_rep1
delCsn2-P_L1_STAT5_rep2
delCsn2-P-E123_L1_GR_rep1
delCsn2-P-E123_L1_H3K27ac_rep1
delCsn2-P-E123_L1_H3K27ac_rep2
delCsn2-P-E123_L1_PolII_rep1
delCsn2-P-E123_L1_PolII_rep2
delCsn2-P-E123_L1_STAT5_rep1
delCsn2-P-E123_L1_STAT5_rep2
delCsn2-P-E123-A_L1_GR_rep1
delCsn2-P-E123-A_L1_GR_rep2
delCsn2-P-E123-A_L1_H3K27ac_rep1
delCsn2-P-E123-A_L1_H3K27ac_rep2
delCsn2-P-E123-A_L1_PolII_rep1
delCsn2-P-E123-A_L1_PolII_rep2
delCsn2-P-E123-A_L1_STAT5_rep1
delCsn2-P-E123-A_L1_STAT5_rep2
delCsn2-P-E123-B_L1_GR_rep1
delCsn2-P-E123-B_L1_GR_rep2
delCsn2-P-E123-B_L1_H3K27ac_rep1
delCsn2-P-E123-B_L1_H3K27ac_rep2

delCsn2-P-E123-B_L1_PolII_rep1
delCsn2-P-E123-B_L1_PolII_rep2
delCsn2-P-E123-B_L1_STAT5_rep1
delCsn2-P-E123-B_L1_STAT5_rep2
delCsn3-E1_L1_NFIB_rep1
delCsn3-E1_L1_PolII_rep1
delCsn3-E1_L1_PolII_rep2
delCsn3-E2-S_L1_GR_rep1
delCsn3-E2-S_L1_GR_rep2
delCsn3-E2-S_L1_H3K27ac_rep1
delCsn3-E2-S_L1_H3K27ac_rep2
delCsn3-E2-S_L1_NFIB_rep1
delCsn3-E2-S_L1_NFIB_rep2
delCsn3-E2-S_L1_PolII_rep1
delCsn3-E2-S_L1_PolII_rep2
delCsn3-E2-S_L1_STAT5_rep1
delCsn3-E2-S_L1_STAT5_rep2
delCsn3-E2-SN_L1_GR_rep1
delCsn3-E2-SN_L1_GR_rep2
delCsn3-E2-SN_L1_H3K27ac_rep1
delCsn3-E2-SN_L1_H3K27ac_rep2
delCsn3-E2-SN_L1_NFIB_rep1
delCsn3-E2-SN_L1_NFIB_rep2
delCsn3-E2-SN_L1_PolII_rep1
delCsn3-E2-SN_L1_PolII_rep2
delCsn3-E2-SN_L1_STAT5_rep1
delCsn3-E2-SN_L1_STAT5_rep2
delOdam_L1_GR_rep1
delOdam_L1_GR_rep2
delOdam_L1_H3K27ac_rep1
delOdam_L1_H3K27ac_rep2
delOdam_L1_NFIB_rep1
delOdam_L1_PolII_rep1
delOdam_L1_PolII_rep2
delOdam_L1_STAT5_rep1
delOdam_L1_STAT5_rep2
delOdam_SG_H3K27ac_rep1
delOdam_SG_H3K27ac_rep2
delOdam_SG_PolII_rep1
delOdam_SG_PolII_rep2
WT_p6_PolII_rep1
WT_p6_PolII_rep2
WT_SG_H3K27ac_rep1
WT_SG_H3K27ac_rep2
WT_SG_PolII_rep1
WT_SG_PolII_rep2
WT_virMEC_H3K27ac
WT_virMEC_NFIB
WT_virMEC_PolII

Genome browser session (e.g. UCSC)

no longer applicable

Methodology

Replicates

delCsn2-P-E123_L1_GR, delCsn3-E1_L1_NFIB and delOdam_L1_NFIB samples have one data set because they were not critical for the study. WT_virMEC_H3K27ac, WT_virMEC_NFIB and WT_virMEC_PolII samples have one data set because several mice were used for one ChIP-seq. For all other ChIP-seq experiments more than two replicates were conducted.

Sequencing depth

All Sequencing was done as 51bp single end sequence. Sequencing was done to achieve > 30 million reads per biological replicate.

Antibodies

TAT5A (Santa Cruz Biotechnology, sc-1081 and sc-271542), GR (Thermo Fisher Scientific, PA1-511A), NFIB (Sigma-Aldrich, HPA003956), MED1 (Bethyl Laboratory, A300-793A), H3K27ac (Abcam, ab4729), RNA polymerase II (Abcam, ab5408), H3K4me1 (Active Motif, 39297) and H3K4me3 (Millipore, 07-473)

Peak calling parameters

MACS2 was used with default settings for peak calling.

Data quality

> 20000 peaks for transcription factors and > 100000 peaks for histone markers by q-value (< 0.001 for TFs, 0.1 or 0.5 for histone markers) were at 5% FDR and above 4-fold enrichment.

Software	FastQC tool (version 0.11.9); Trimmomatic (version 0.36);Bowtie (version 1.2.2); Samtools (version 1.8); Picard; Homer (version 4.9.1); DeepTools (version 3.1.3); IGV(version 2.5.3); MACS (version 2.2.7.1); Bedtools (version 2.29.2)
